# Mechanisms of NK Cell-Macrophage *Bacillus anthracis* Crosstalk: A Balance between Stimulation by Spores and Differential Disruption by Toxins

**DOI:** 10.1371/journal.ppat.1002481

**Published:** 2012-01-12

**Authors:** Maria Klezovich-Bénard, Jean-Philippe Corre, Hélène Jusforgues-Saklani, Daniel Fiole, Nick Burjek, Jean-Nicolas Tournier, Pierre L. Goossens

**Affiliations:** 1 Laboratoire Pathogénie et Toxi-Infections Bactériennes, Institut Pasteur, Paris, France; 2 CNRS URA 2172, Paris, France; 3 Unité d'Immunobiologie des Cellules Dendritiques, Institut Pasteur, Paris, France; 4 Unité Interactions Hôte-Agents Pathogènes, Département de Microbiologie, Institut de Recherche Biomédicale des Armées, La Tronche, France; 5 Laboratoire Interdisciplinaire de Physique, UMR 5588 CNRS/Université Joseph Fourier, St-Martin-d'Hères, France; 6 École du Val-de-Grâce, Paris, France; University of California Los Angeles, United States of America

## Abstract

NK cells are important immune effectors for preventing microbial invasion and dissemination, through natural cytotoxicity and cytokine secretion. *Bacillus anthracis* spores can efficiently drive IFN-γ production by NK cells. The present study provides insights into the mechanisms of cytokine and cellular signaling that underlie the process of NK-cell activation by *B. anthracis* and the bacterial strategies to subvert and evade this response. Infection with non-toxigenic encapsulated *B. anthracis* induced recruitment of NK cells and macrophages into the mouse draining lymph node. Production of edema (ET) or lethal (LT) toxin during infection impaired this cellular recruitment. NK cell depletion led to accelerated systemic bacterial dissemination. IFN-γ production by NK cells in response to *B. anthracis* spores was: i) contact-dependent through RAE-1-NKG2D interaction with macrophages; ii) IL-12, IL-18, and IL-15-dependent, where IL-12 played a key role and regulated both NK cell and macrophage activation; and iii) required IL-18 for only an initial short time window. *B. anthracis* toxins subverted both NK cell essential functions. ET and LT disrupted IFN-γ production through different mechanisms. LT acted both on macrophages and NK cells, whereas ET mainly affected macrophages and did not alter NK cell capacity of IFN-γ secretion. In contrast, ET and LT inhibited the natural cytotoxicity function of NK cells, both *in vitro* and *in vivo*. The subverting action of ET thus led to dissociation in NK cell function and blocked natural cytotoxicity without affecting IFN-γ secretion. The high efficiency of this process stresses the impact that this toxin may exert in anthrax pathogenesis, and highlights a potential usefulness for controlling excessive cytotoxic responses in immunopathological diseases. Our findings therefore exemplify the delicate balance between bacterial stimulation and evasion strategies. This highlights the potential implication of the crosstalk between host innate defences and *B. anthracis* in initial anthrax control mechanisms.

## Author Summary

NK cells are important immune effectors that perform a surveillance task and react to transformed, stressed, and virally infected cells. They represent a first-line defence against cancer and pathogen invasion. Different pathogens trigger distinct NK-cell activation pathways. The *Bacillus anthracis* spore is the highly resistant form that enters the host and provokes anthrax. This microbe kills through a combination of acute bacterial infection and devastating toxemia. In the present study, we characterise the crosstalk between NK cells and spores, as well as the strategies used by *B. anthracis* to evade initial control mechanisms and impact anthrax pathogenesis. Our findings exemplify the spores' property to efficiently drive a high production of IFN-γ by NK cells, as well as the complex pathways used for activation which require both cytokine and cellular signaling. *B. anthracis* subverts this response through its toxins by paralysing essential NK cell functions. Furthermore, edema toxin from *B. anthracis* blocks natural cytotoxicity without affecting IFN-γ secretion. The CyaA toxin of *Bordetella pertussis* possesses the same enzymatic activity and has a similar effect. The high efficiency of these toxins in blocking cytotoxicity *in vivo* implies possible exploitation of their subverting activity to modulate excessive cytotoxic responses in immunopathological diseases.

## Introduction

NK cells are immune cells that do not need prior exposure to antigen to exert their functions. Their receptors are germline encoded and do not require somatic gene rearrangements. These receptors recognise an array of self-molecules through highly specific mechanisms. The functions of NK cells are regulated through a delicate balance between activating and inhibitory receptors. Although NK cells are traditionally considered as belonging to the innate immune system, a number of recent reports have shown that NK cell education can occur, leading to an expansion of pathogen-specific cells and generation of ‘memory’ cells [1]. NK cells perform a surveillance task and react to transformed, stressed, and virally infected cells. They represent a first-line of defence against cancer and pathogen invasion.

NK cells are important immune effectors for preventing microbial invasion and dissemination [1]. They are found in blood as well as in peripheral nonlymphoid tissues and secondary lymphoid organs [1]. In early host responses, NK cells exert two principal functions: secretion of a range of cytokines and natural cytotoxicity. Among secreted cytokines, IFN-γ plays a key role in enhancing immune responses, in particular by modulating macrophage activation [2]. NK-cell activation is readily induced during viral and bacterial infections and requires cytokine and receptor signals that are delivered by myeloid cells [3]–[5], such as IFN-α/β [6], IL-12 [7], IL-15 [6] and IL-18 [8]. Apart from a potential role in polymicrobial sepsis [9], NK-cell implication during bacterial infections has been studied in few models, mainly of intracellular bacteria (*Mycobacterium sp.*, *Listeria monocytogenes*, *Salmonella enterica* serovar *Typhimurium*) [5]. Among the extracellular bacteria, *Staphylococcus aureus* and the anaerobe *Lactobacillus johnsonii* have been reported to stimulate NK cells [5].

Spores of the extracellular bacterial pathogen *Bacillus anthracis* can efficiently drive IFN-γ production in large amounts by NK cells [10]. The spore is the infectious bacterial form that first interacts with the host, thereby eliciting the earliest host defences against infection. The innate immune response was originally considered as a non-specific response characterized by engulfment and digestion of microorganisms and foreign substances by phagocytic cells. However, innate immunity does show considerable specificity through the activation of different signaling pathways associated with different Toll-like receptors (TLRs) that recognise different pathogen-associated molecular patterns. Activation of TLRs thus results in different biological responses depending on the pathogen. NK cell activation induced by the *B. anthracis* spore is independent of TLR2, TLR4, and TLR7 [10] and is probably dependent on multiple receptor engagement due to the complex nature of the spore. The downstream signaling pathways nevertheless implicate the adaptor molecule MyD88 [10]. Among the primary effector cells of innate immunity to intervene at the portal of *B. anthracis* entry, macrophages have been implicated in two contrasting processes: (i) the initiation of infection by playing a role in spore germination [11] and spore dissemination [12], and (ii) the control of infection through spore phagocytosis and destruction [11], [13], [14]. Neutrophils are recruited in the first hours of infection and are involved in the early control of infection [14], [15]. Lung dendritic cells (DCs) play a pivotal role in spore uptake and promote dissemination of spores from the alveolar space into the draining lymph nodes [14].

Many of the symptoms of systemic anthrax can be attributed to the subversive effects of a poly-γ-D-glutamic capsule and toxins. Anthrax toxins suppress the immune defences of the host by targeting cells of innate and adaptive immunity. *B. anthracis* thus completely evades almost all of the key players responsible for efficient host protection. *B. anthracis* toxins are made up of three secreted proteins, protective antigen (PA), edema factor (EF; a calmodulin-dependent adenylate cyclase that increases intracellular concentrations of cAMP in host cells [16]) and lethal factor (LF; a zinc-dependent metalloprotease which cleaves the N-terminal region of mitogen-activated protein kinase kinases [MAPKKs] [17]). Functionally, the combination of PA with EF or LF forms edema toxin (ET) or lethal toxin (LT) respectively [18], [19]. LT kills or inactivates monocytes, macrophages, and neutrophils [14] whereas ET disrupts cytokine networks in monocytes [14], and reduces macrophage migration [14]. Both LT and ET impair neutrophil actin-based motility, resulting in paralysis of PMN chemotaxis [14], [20]. Anthrax toxins impair activation and maturation of DCs, thus blocking the initiation of adaptive immunity [21], [22]. LT disrupts TCR signaling in CD1d-restricted NKT cells, leading to functional unresponsiveness [23]. LT also severely reduces B-cell proliferation, impairs immunoglobulin production [24], and blocks MAPKK-dependent cytokine production in CD4+ T cells [25], [26]. Furthermore, ET reduces T-cell migration [14] and disrupts T-cell function [14]. Anthrax toxins are therefore involved in mediating immune evasion of the bacterium by interfering with the innate and adaptive immune responses.

NK cells are pivotal in the first-line of innate defence, serving as a functional bridge between innate and acquired immunity. To our knowledge, their role during *B. anthracis* infection has yet to be addressed. The present study aimed to characterise the mechanisms of NK-cell activation by *B. anthracis* spores, the strategies employed by the bacteria to subvert and evade this response through toxin secretion. We also aimed to identify the relevance of these processes for controlling *B. anthracis* infection. Relatively few studies have linked specific cytokines with protection against *B. anthracis*. However, IFN-γ increases the ability of macrophages to resist destruction by *B. anthracis* and to kill the bacteria [27] and we have shown that sensitivity to *B. anthracis* infection was partially dependent on IFN-γ [28]. As NK cells are the main innate immune cells to produce IFN-γ, we focused our study on these novel cellular targets of anthrax toxins in the context of the complex heterocellular interactions induced by *B. anthracis* spores.

## Results

### *B. anthracis* spores activate NK cells through accessory-cell- and contact-dependent interactions

**Figure 1:**
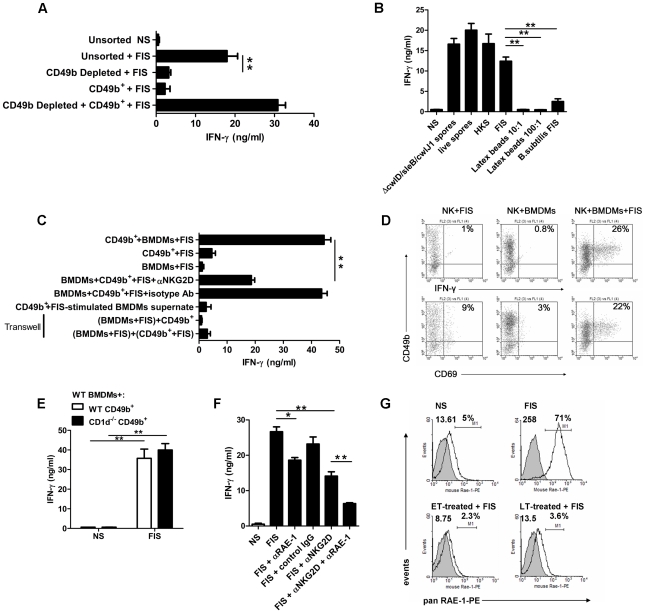
Contact dependence of NK cell activation by *B. anthracis* spores. C57BL/6 splenocytes, either unsorted (**A** and **B**), fractionated with anti-CD49b magnetic beads (**A**), or purified CD49b^+^ spleen cells cocultured with BMDMs (**C**) were stimulated with formaldehyde-inactivated spores (FIS), or (**B**) with heat-inactivated spores (HKS), live spores in the presence of antibiotics, a germination-deficient strain, formaldehyde-inactivated *B.subtilis* spores or latex beads; (**C**) the NKG2D receptor was neutralized, or the CD49b^+^ cells and BMDMs were separated in a Transwell system. IFN-γ concentrations were determined in the supernatants by ELISA as described in Materials and Methods. Results shown are the mean ± SD for triplicate cultures representative of at least three experiments. (**D**) FACS analysis of surface CD69 and intracellular IFN-γ expression in spore-stimulated CD49b^+^ cells in the presence of BMDMs (see Figure S1A for gating strategy and controls); percentage for each positive cell population shown in the upper right quadrant of each dot plot; data representative of three independent experiments; the mean fold increase was 8.4±0.7 and 12.4±5 for CD69 and IFN-γ expression respectively; (**E**) CD49b^+^ cells from NKT-deficient CD1d^−/−^ mice stimulated with FIS in the presence of BMDMs from WT C57BL/6 mice produce similar amounts of IFN-γ than WT C57BL/6 mice; (**F**) same experimental conditions as in (**C**) with neutralization of NKG2D and/or RAE-1. (**G**) FACS analysis of RAE-1 surface expression on FIS-stimulated BMDMs and effect of incubation with ET and LT; mean fluorescence intensity of the positive peak (upper left corner) and percentage of the positive cell population are shown in each dot plot. Isotype-matched controls Abs were used for each staining combination (filled histogram). Data are representative of at least three independent experiments giving similar results. Significant differences between experimental conditions are indicated with asterisks (t test; *, *P*<0.05; **, *P*<0.01).

*B. anthracis* spores induce naive spleen cells to secrete large amounts of IFN-γ. This phenomenon depends upon the interactions between CD49b^+^ cells and accessory cells from the CD49b-negative fraction (Figure 1A). Depletion of CD49b^+^ cells led to a significant 6.5-fold decrease of IFN-γ production (Figure 1A). Direct stimulation of purified CD49b^+^ cells with spores did not induce IFN-γ secretion. However, co-culture of positively selected CD49b cells with CD49b-negative splenocytes restored IFN-γ production upon spore stimulation (Figure 1A).

As our study was focused on spore components as the initial stimuli of the innate immune system, spores needed to be inactivated and prevented from germinating and developing into vegetative bacilli. Different means of inactivation (i.e. heat-inactivation, germination-deficient spores, and live spores in the presence of antibiotics) did not modify the extent of IFN-γ induction when compared with formaldehyde inactivation (Figure 1B). Spores of the phylogenetically distant *B. subtilis* gave rise only to a modest IFN-γ production, demonstrating that *B. anthracis* spore components are more efficient in inducing IFN-γ production than those of *B. subtilis*. Latex beads of comparable size as *B. anthracis* spores were ineffective in stimulating IFN-γ secretion by splenocytes, indicating that phagocytosis of inert particles alone was not sufficient and that *B. anthracis* spores were central for this response.

Similarly, stimulation with spores of positively selected CD49b cells with bone-marrow derived macrophages (BMDMs) resulted in a strong induction of IFN-γ secretion (Figure 1C) and activation of CD49b^+^ cells (Figure 1D); *i.e.* a 7.3-fold upregulation of the leukocyte early activation marker CD69 and a 32-fold increase of intracellular IFN-γ positive cells (Figure 1D and S1A). Stimulation of isolated CD49b^+^ cells or BMDMs by spores did not result in IFN-γ secretion (Figure 1C), nor did it change basal expression of CD69 or intracellular IFN-γ (Figure 1D). IFN-γ secretion by CD49b^+^ cells from NKT-deficient CD1d^−/−^ mice [29], [30] was similar to that by CD49b^+^ wild-type cells when co-cultured with spore-stimulated wild-type BMDMs (Figure 1E). This strongly suggests that NK cells were the main source of IFN-γ production in this bacterial system.

NK-cell activity is regulated by both contact-dependent and soluble signals. Direct contact between BMDMs and NK cells was necessary for NK cell activation as: (i) conditioned medium from spore-stimulated BMDMs did not elicit IFN-γ secretion (Figure 1C); and (ii) physical separation of purified NK cells and BMDMs in compartments of transwell plates did not lead to IFN-γ secretion, even if stimulation with the spores was effected in both compartments (Figure 1C). The activating receptor, NKG2D, elicits cytokine production by NK cells [31], [32]. The present study also implicated the NKG2D receptor in the interaction between NK cells and spore-stimulated BMDMs as addition of neutralizing antibody for the NKG2D receptor decreased IFN-γ production (Figure 1C and 1F). Similarly, neutralizing antibody for RAE-1 —one of the NKG2D ligands— partially inhibited IFN-γ production and co-neutralization of NKG2D and RAE-1 led to a greater reduction (Figure 1F). Finally, stimulation of BMDMs with *B. anthracis* spores resulted in an approximately 19-fold upregulation of RAE-1 expression compared with non-stimulated BMDMs (Figure 1G). The activating receptor NKp46 was not involved as its neutralization did not modify IFN-γ secretion induced by spores (Figure S1B).

Taken together, these results indicate that NK cell activation and IFN-γ production in response to *B. anthracis* spores are dependent on an accessory cell, are contact-dependent, and occur through RAE-1-NKG2D interaction.

### Functional relationship between IL-12 and IL-18 during stimulation by *B. anthracis* spores

**Figure 2:**
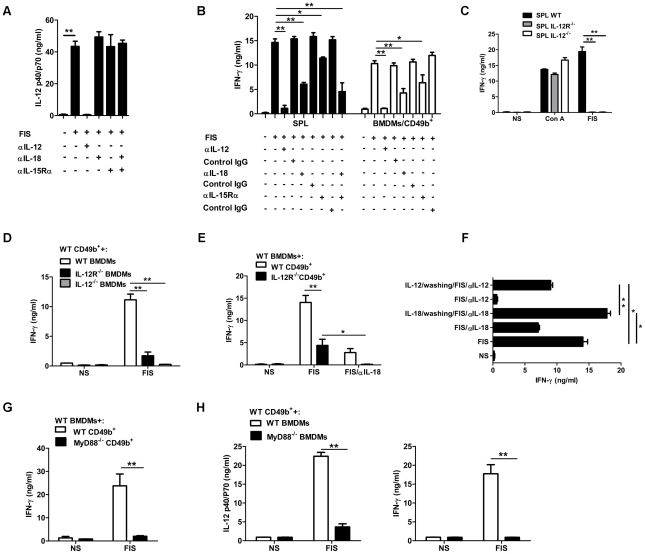
Network of cytokine dependence of NK cell activation by *B. anthracis* spores. Effect of neutralization of IL-12, IL-18 or IL-15Rα on (**A**) IL-12p40/p70 concentration in culture supernatants of FIS-stimulated BMDMs or (**B**) IFN-γ production by splenocytes (SPL; left panel), or purified CD49b^+^ cells co-cultured with BMDMs (right panel). (**C**) Splenocytes (SPL) from wild-type (WT), IL-12R^−/−^ or IL-12^−/−^ C57BL/6 mice were stimulated with FIS, or ConA as a positive control. (**D**) CD49b^+^ cells from WT C57BL/6 mice were co-cultured with BMDMs from IL-12R^−/−^ or IL-12^−/−^ C57BL/6 mice in the presence of FIS. (**E**) CD49b^+^ cells from WT or IL-12R^−/−^ C57BL/6 mice were co-cultured with BMDMs from WT C57BL/6 mice in the presence of FIS with or without IL-18 neutralizing antibody. (**F**) Effect of short-term priming with IL-12 or IL-18 on spore-stimulation of splenocytes; corresponding cytokine neutralization was maintained for the remainder of the assay. (**G**) Purified CD49b^+^ cells from WT or MyD88^−/−^ C57BL/6 mice were co-cultured with BMDMs from WT C57BL/6 in the presence of FIS. (**H**) Purified CD49b^+^ cells from C57BL/6 WT mice were co-cultured with BMDMs from WT or MyD88^−/−^ C57BL/6 mice in the presence of FIS; IL-12 (left panel), or IFN-γ (right panel) production. For all experiments with purified CD49b^+^ cells, no IFN-γ was detected after direct stimulation with spores (**D, E, G, H**; data not shown). For all experiments, values are mean ± SD for at least three measurements and are representative of at least three independent experiments. Significant differences between experimental conditions are indicated with asterisks (t test; *, *P*<0.05; **, *P*<0.01).

Stimulation of BMDMs with *B. anthracis* spores induced secretion of IL-12 (Figure 2A). Neutralization of IL-18 or blocking the IL-15Rα receptor did not alter IL-12 secretion. Macrophage-secreted IL-12 appeared to play a key role in NK-cell activation by *B. anthracis* spores as: (i) IL-12 neutralizing antibodies abolished IFN-γ secretion both in splenocytes and co-cultured BMDMs/purified NK cells (Figure 2B), and (ii) stimulation of splenocytes from IL-12^−/−^ and IL-12 R^−/−^ mice did not elicit IFN-γ production (Figure 2C). Furthermore, absence of IL-12 secretion by spore-stimulated BMDMs from IL-12^−/−^ mice did not induce IFN-γ secretion by wild-type NK cells (Figure 2D).

However, NK cells from IL-12R^−/−^ mice were weakly activated by spore-stimulated wild-type BMDMs (Figure 2E), indicating that other signals besides IL-12 were able to activate NK cells. This low amount of IFN-γ production was completely abolished by the addition of neutralizing antibody for IL-18 (Figure 2E). Thus, in the absence of IL-12 signaling, IL-18 becomes essential for the induction of IFN-γ. This was experimentally confirmed by the addition of IL-18 neutralizing antibodies, which significantly decreased spore-induced IFN-γ production by wild-type splenocytes or co-cultured BMDMs and purified NK cells (Figure 2B). Expression of the IL-12 receptor by BMDMs was also important for full NK cell activation in response to spores; IFN-γ production by wild-type NK was significantly lower with spore-stimulated BMDMs from IL-12R^−/−^ mice compared with their wild-type counterparts (Figure 2D). This finding implies a positive feedback of IL-12 and its important role in influencing the stimulatory capacities of macrophages.

Blocking the IL-15Rα receptor led to a partial but significant inhibition of IFN-γ production in both spore-stimulated splenocytes and purified NK cells co-cultured with BMDMs (Figure 2B). Finally, simultaneous neutralization of IL-18 and IL-15 strongly decreased IFN-γ production (Figure 2B). This indicates that IL-12-induced IFN-γ production depends upon IL-18 and IL-15 and suggests a synergistic mechanism of action of IL-12 with IL-18 and/or IL-15. As mentioned above, inhibition of IFN-γ production by IL-18 or/and IL-15 neutralizing antibodies was not associated with a decrease in IL-12 secretion (Figure 2A). The hypothesis that IL-18 or IL-15 are necessary for optimal IL-12 secretion by spore-stimulated BMDMs can therefore be excluded. Of the other cytokines tested, TNF-α, IFN-α, IFN-β, and IL-10 were not involved in the stimulation of IFN-γ secretion, as their neutralization did not interfere with IFN-γ secretion (Figure S1B).

IL-18 is stored in internal cellular compartments as a precursor and can rapidly be secreted through caspase-1 activation whereas IL-12 needs to be synthesized before secretion. To explore whether IL-18 is secreted earlier than IL-12 in spore-induced macrophage activation and NK cell IFN-γ production, we determined whether short-term priming with IL-18 or IL-12 was sufficient to achieve full activation of NK cells during stimulation with *B. anthracis* spores. A 4-h priming of splenocytes with IL-18 before spore stimulation followed by neutralization of IL-18 for the remaining incubation period with spores was sufficient to reach similar concentrations of IFN-γ to those obtained in the absence of IL-18 neutralization. In contrast, IL-18 neutralization for the entire length of spore-incubation significantly decreased IFN-γ production (Figure 2F). However, similar short-term priming with IL-12, followed by neutralization of IL-12 for the remaining incubation period with spores, only partially restored the IFN-γ response compared with spore-incubation without IL-12 neutralization (Figure 2F). Thus *B. anthracis* spores induce IL-18 signaling as a primary event that probably synergizes with IL-12 signaling which needs to be effective for a longer period to activate IFN-γ secretion by NK cells. Both IL-12 and IL-18 are therefore essential for IFN-γ production by NK cells in response to *B. anthracis* spores.

Spore recognition by macrophages triggers downstream signaling pathways that are dependent on MyD88, an adapter protein essential not only for the induction of inflammatory cytokines triggered by TLRs, but also for signaling downstream to IL-18 and IL-1 receptors. Splenocytes from MyD88-deficient mice cannot produce IFN-γ in response to *B. anthracis* spores [10]. To identify the cells on which the MyD88 adapter protein is required, we performed mixed experiments co-culturing macrophages and NK cells from WT or MyD88-deficient mice. NK cells deficient in MyD88 did not produce IFN-γ; this is most likely due to the defect in IL-18 downstream signaling as IL-18 is central in this system of NK cell activation (Figure 2G). Macrophages from MyD88-deficient mice were also impaired in their capacity to help NK cells to produce IFN-γ following spore stimulation. IL-12 production was strongly decreased (Figure 2H, left panel), thus inhibiting IFN-γ production (Figure 2H, right panel). These results demonstrate that MyD88 is implicated both in the macrophage —through recognition of *B. anthracis* spores by yet to be characterised pathogen pattern-based receptors— and in the NK cells —through activation of the IL-18 signaling pathway.

### Dissociation of NK cell functions by ET *versus* global inhibition by LT

As *B. anthracis* toxins secreted by the nascent bacilli upon spore germination could subvert the innate immune response to spores at the initial step of infection, the effects of toxins on spore-induced IFN-γ production were evaluated.

The toxins interfered with contact-dependent signaling, as the spore-induced increase in RAE-1 expression on BMDMs was efficiently downregulated by ET or LT-treatment (Figure 1G).

**Figure 3:**
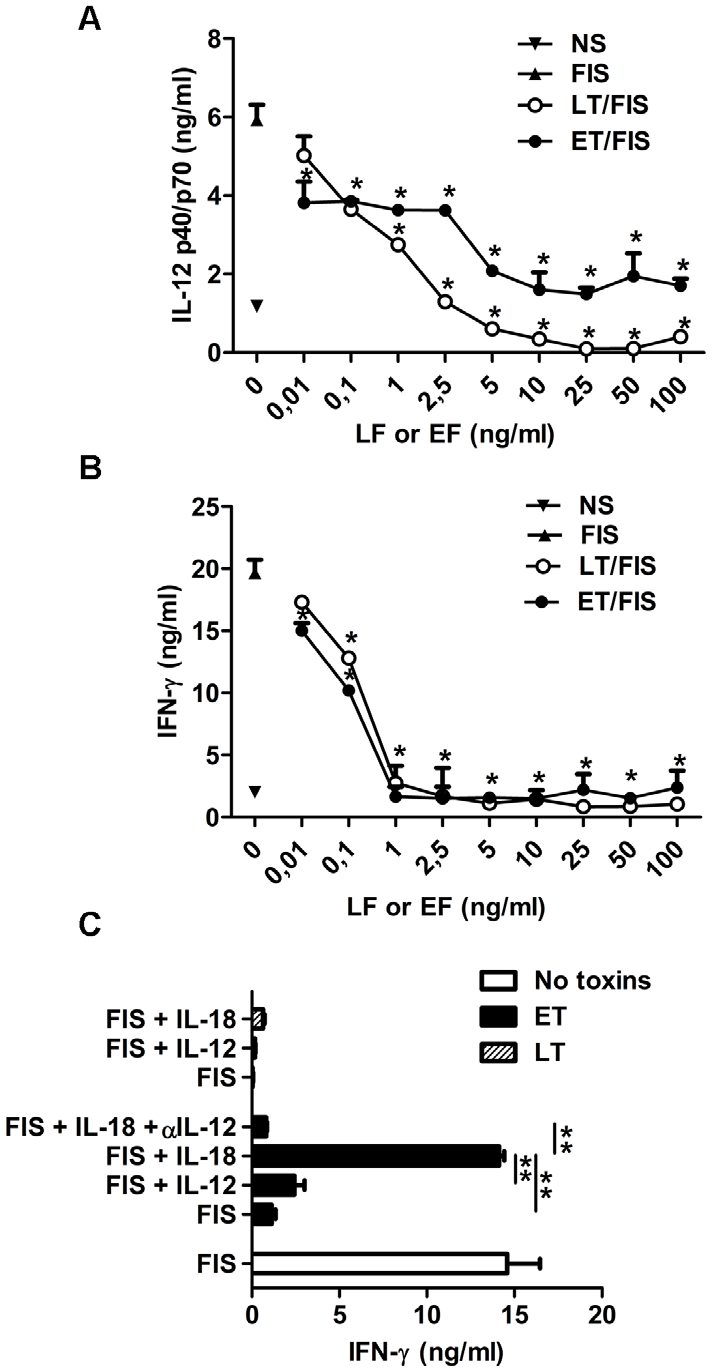
Differential inhibition by ET and LT of the spore-induced IL-12 and IFN-γ production by splenocytes. IL-12p40/p70 (**A**) and IFN-γ (**B**) production by splenocytes pre-incubated for 1 h with PA and increasing concentrations of LF or EF; spore stimulation was then performed as in Figure 1A in the presence of toxins. (**C**) Similar incubation conditions as in (**A,B**) with either addition of rIL-18 or rIL-12p70, or IL-12 neutralization. The data represent mean cytokine concentrations of triplicates in culture supernatants (± SD) representative of three independent experiments. T test; *, *P*<0.05 compared with the group incubated with FIS without toxins.

The toxins also impaired cytokine-dependent signaling. LT disrupted the ability of splenocytes stimulated with *B. anthracis* spores to produce both IL-12 and IFN-γ, in a dose-dependent manner (Figure 3A and 3B); doses as low as 1 ng/ml and 0.1 ng/ml, respectively, significantly inhibited IL-12 and IFN-γ secretion. The addition of IL-12 or IL-18 did not restore LT-inhibited IFN-γ secretion (Figure 3C). Cell viability was unaffected (Figure S1C). These results suggest that LT blocks the NK cell response to IFN-γ inducing stimuli by interfering with IL-12 and IL-18 signaling.

**Figure 4:**
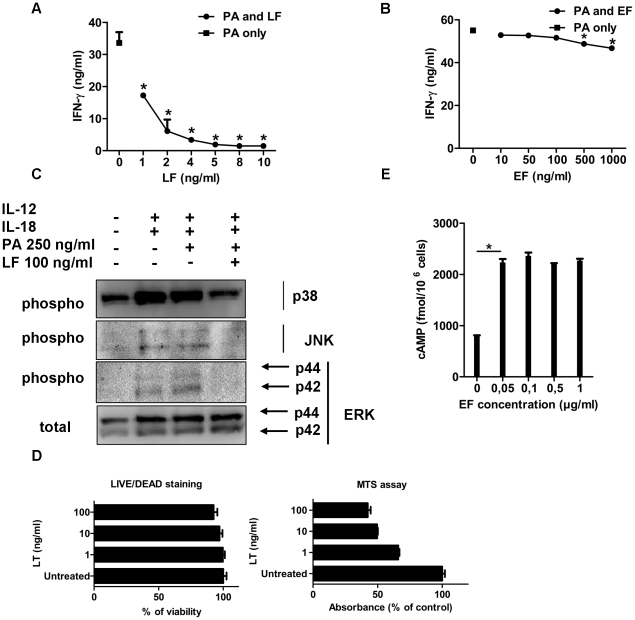
Impairment of IFN-γ production by LT in purified NK cells, contrasting with absence of effect by ET. (**A,B**) IFN-γ production by purified CD49b^+^ cells pre-treated for 1 h with PA and increasing concentrations of LF or EF and then stimulated for the whole incubation time with rIL-12 and rIL-18 in the presence of toxins. Data are mean ± SD of triplicates and are representative of one experiment of three performed; SD values are hidden by symbol size. T test; *, *P*<0.05 compared with the group incubated with PA only. (**C**) Inhibition of p38, JNK and ERK phosphorylation by LT in purified CD49b+ cells activated by rIL-12 and rIL-18 for 10 min; total ERK1/2 was used as loading control. Data represent one of at least two independent experiments. (**D**) NK cell viability (left panel; Live/Dead Cell Staining) and metabolic activity (right panel; MTS assay) after 18 h-incubation with LT. *, *P*<0.05 compared to the untreated group. (**E**) Intracellular cAMP production by purified CD49b^+^ cells treated with ET for 1 h. Data are mean ± SD of triplicates per condition and are representative of one experiment out of three. T test; *, *P*<0.05 compared with the untreated group.

To explore the direct effects of LT on NK cells and the mechanisms of inhibition of IFN-γ production by LT, purified NK cells were co-stimulated with IL-12 and IL-18 in the absence of accessory cells. As expected [33], this resulted in abundant IFN-γ production (Figure 4A and 4B; PA only) and rapid phosphorylation of p38, ERK1/2, and JNK MAPK (Figure 4C). IFN-γ secretion by IL-12/IL-18-stimulated purified NK cells was totally inhibited with LT in a dose-dependent manner (Figure 4A); statistically significant inhibition was observed from doses as low as 1 ng/ml in these conditions of strong stimulation. LT inhibited p38, ERK1/2, and JNK MAPK phosphorylation (Figure 4C). The viability of NK cells was unaffected by LT treatment, even at the highest dose of 100 ng/ml over the 18 h culture period (Figure 4D). Assessment of metabolic activity using the MTS assay (which measures the activity of mitochondrial deshydrogenases) showed a reduction at 1 ng/ml and reached a plateau from 10 ng/ml with a *circa* 50% decrease (Figure 4D). Metabolic activity was thus low in LT-treated NK cells, whereas viability was maintained.

**Figure 5:**
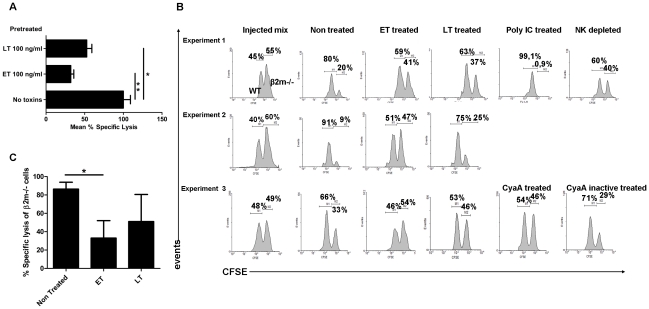
ET efficiently inhibits NK cell cytotoxic activity *in vitro* and *in vivo*. (**A**) Pre-incubation of purified CD49b^+^ cells with ET and LT inhibits lysis of YAC-1 target cells. Data represent mean ± SD (n = 3) of one of at least three independent experiments. T test; *, *P*<0.05, ** *P*<0.01 as compared with the no-toxin group. (**B**) *In vivo* effect of ET and LT on the natural cytotoxic activity of NK cells: C57BL/6 wild-type and syngeneic MHC class I-deficient β2m−/− splenocytes were differentially labeled with CFSE and adoptively transferred intravenously in equal number (“injected mix”) into C57BL/6 syngeneic wild-type recipients; elimination of the MHC class I-deficient cells (CFSE high) was quantified 16–20 h later in the spleen and confirmed to be mediated by the NK cell population of the recipients, either after *in vivo* NK cell activation by poly:(IC) injection, or after *in vivo* NK cell depletion through injection of anti-NK1.1 antibodies (experiment 1). The effect on elimination of the MHC class I-deficient cells of ET, LT (experiments 1 to 3) or the toxin CyaA of *Bordetella pertussis* (or its inactive mutant CyaE5) (experiment 3) was then quantified: all toxins were injected intravenously 8 h prior CFSE-labeled mixed cell inoculation. Controls were injected with PA, EF, or LF only; MHC class I-deficient cells were eliminated as in the non-treated recipients (Figure S1D). Data represent histogram plots from three independent experiments showing relative percentages of the high (MHC class I-deficient) and low (normal) CFSE cell populations. (**C**) Mean percent specific lysis of MHC class I-deficient cells of 3 independent assays performed. The percent specific lysis was calculated as described in Materials and Methods. T test; *, *P*<0.05 compared to the untreated group.

NK cells possess a dual function, cytokine secretion and natural cytotoxicity towards specific targets [1]. We determined whether LT could also alter the cytotoxic activity of NK cells. LT significantly reduced the ability of NK cells to kill the mouse YAC-1 lymphoma target cell *in vitro* (Figure 5A). Thus, our results show that NK cells are highly sensitive to LT subverting both functions, leading to a strong inhibition of cytokine secretion and natural cytotoxicity.

Similarly, ET disrupted the ability of splenocytes stimulated with *B. anthracis* spores to produce both IL-12 and IFN-γ in a dose-dependent manner (Figure 3A and 3B). A dose of 10 pg/ml significantly inhibited both IL-12 and IFN-γ secretion, whereas the basal secretion IL-12 was not inhibited (Figure 3A). The addition of IL-12 during spore stimulation did not restore the IFN-γ secretion (Figure 3C). In contrast, the ET-mediated inhibition was reversed by addition of IL-18 (Figure 3C). This was related to the persistence of a basal IL-12 secretion, as addition of neutralizing antibodies for IL-12 abolished restoration of IFN-γ production by recombinant IL-18 (Figure 3C). These results strongly suggest that ET blocked IFN-γ production by acting mainly on macrophages, as NK cells were still functional and able to secrete IFN-γ.

To explore in detail the direct effects of ET on IFN-γ secretion inhibition in NK cells, IL12/18-stimulated purified NK cells were exposed to graded doses of ET in the absence of accessory cells. ET did not inhibit IFN-γ production at doses up to 100 ng/ml, and only slightly at the high dose of 1 µg/ml (Figure 4B), despite expression of adenylate cyclase activity, detected by a marked increase in intracellular cAMP (2.8-fold higher than in untreated IL-12/IL-18-stimulated purified NK cells) (Figure 4E). In contrast, ET significantly reduced the ability of NK cells to kill the YAC-1 target cells *in vitro*, exhibiting an even stronger inhibition capacity when compared with the effects of a similar dose of LT (Figure 5A).

The above results show that: (i) ET and LT similarly disrupt IFN-γ production by spore-stimulated splenocytes, but the mechanism of inhibition is different for each toxin; LT acts both on macrophages and NK cells, whereas ET acts on macrophages, and (ii) both ET and LT directly alter the innate ability of NK cells to exert their natural cytotoxicity function.

During anthrax infection, *B. anthracis* toxins are present in the infected tissues and circulate in the host vascular system. To evaluate the functional consequences on the natural cytotoxic activity of NK cells *in vivo*, we exploited an *in vivo* model of NK cell cytototoxic activity relying on the capacity of NK cells to recognise and eliminate MHC class I-deficient cells. Equal ratios of MHC class I-deficient splenocytes (from C57BL/6 β2m^−/−^, high CFSE labeling) and wild-type C57BL/6 MHC class I-expressing splenocytes (low CFSE labeling) (Figure 5B; “injected mix”) were injected intravenously into syngeneic wild-type C57BL/6 mice. The elimination of the MHC class I-deficient β2m^−/−^ cells was quantified in the spleen relative to the MHC class I-expressing cells [34]. In this *in vivo* model, MHC class I-deficient β2m^−/−^ cells are rapidly eliminated 16–24 h following adoptive transfer, through the natural cytotoxicity function of NK cells (Figure 5B and 5C; non-treated; cytotoxicity of 94%). As controls, *in vivo* activation of NK cells by injection of the classical NK cell cytotoxic activator poly:(IC) [35] led to elimination of the MHC class I-deficient cells (CFSE high) (Figure 5B). In contrast, *in vivo* depletion of NK cells led to persistence of MHC class I-deficient cells (Figure 5B).

Pretreatment of mice with ET (EF+PA) by intravenous inoculation drastically inhibited clearance of the MHC class I-deficient cells (CFSE high) (Figure 5B and 5C; remaining cytotoxicity of 27%). This effect was related to the adenylate cyclase activity of EF, as the *Bordetella pertussis* toxin CyaA, which has a similar enzymatic activity, also inhibited the elimination of the MHC class I-deficient cells (Figure 5B). EF, LF or PA alone, and a mutated enzymatically inactive CyaA did not modify the capacity of NK cells to specifically eliminate the MHC class I-deficient cells (Figure 5B and S1D). LT (LF+PA) also decreased MHC class I-deficient cell clearance, albeit to a lesser extent (Figure 5B; remaining cytotoxicity of 51%). These results demonstrate the high efficiency of *B. anthracis* toxins, especially ET, in impairing the natural cytotoxicity of NK cells *in vivo*.

### CD49b^+^ and F4/80^+^ cells are rapidly recruited to the draining lymph nodes upon infection with *B. anthracis*

**Figure 6:**
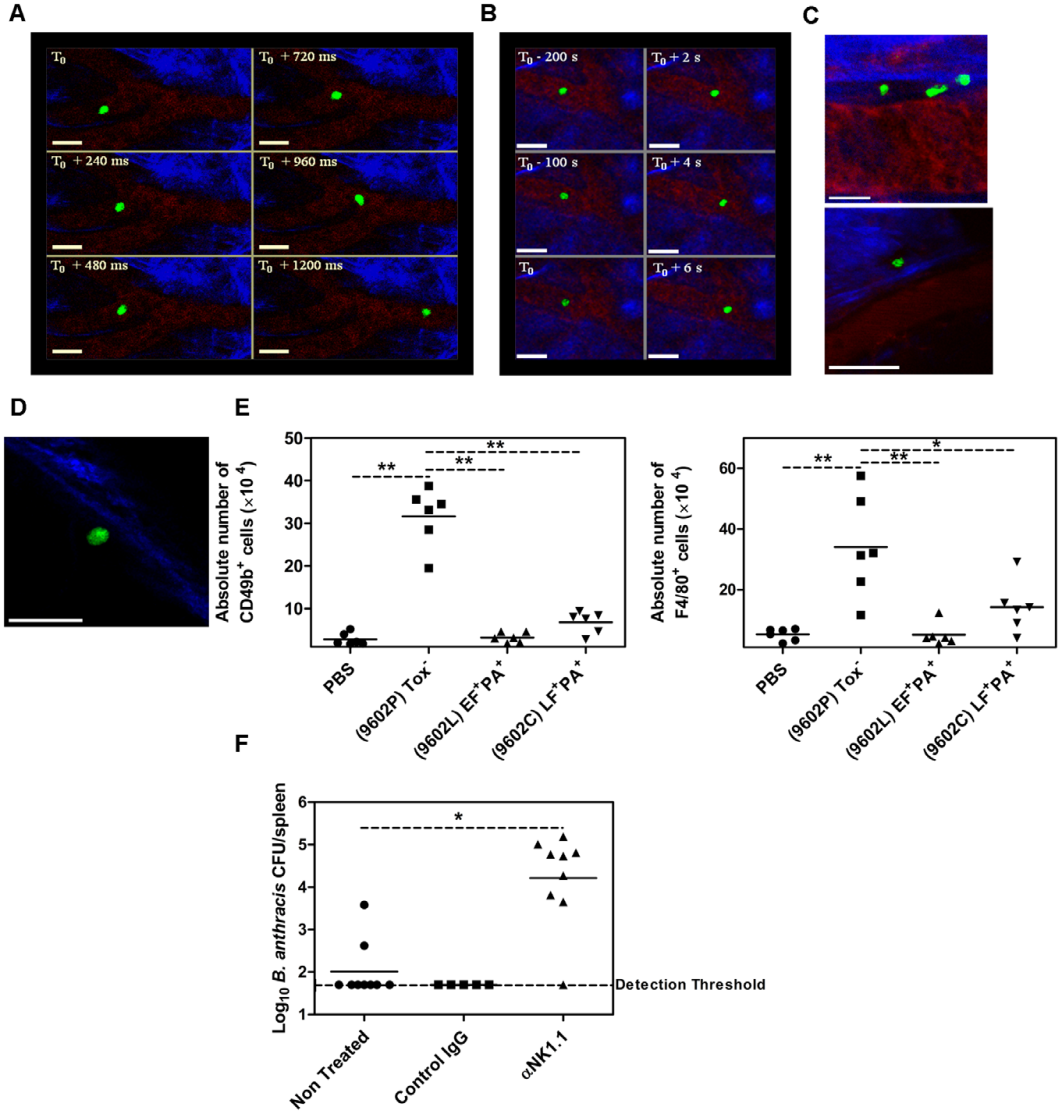
Recruitment and role of NK cells during *B. anthracis* infection and impact of *in vivo* toxin production. (**A**) Circulating NK cells 5 h post-inoculation, viewed by biphoton imaging; dermal collagen in blue (SHG), vascular flow in red (rhodamine B) and NK cells in green (CFSE); scale bar = 20 µm; time-scale in milliseconds indicated on each image. (**B**) Adherent, then rolling NK cell 5 h post-inoculation; scale bar = 20 µm; time-scale in seconds indicated on each image. (**C**) Extravasated NK cells at 18 h post-inoculation; scale bar = 10 µm (top), 40 µm (bottom). (**D**) Subcapsular NK cell in the cervical lymph node draining the infected ear 18 h post-inoculation; NK cells in green (CFSE) and capsular collagen in blue (SHG); scale bar = 20 µm. Data representative of 3 mice. (**E**) Absolute numbers of CD49b^+^ (left panel) and F4/80^+^ cells (right panel) in the cervical lymph node draining the site of cutaneous infection with spores of the 9602P(PA−EF+LF+), 9602L(PA+EF+LF−), 9602C(PA+EF−LF+) strains 24 h post-inoculation (2.91±0.03 log_10_ CFU per mouse). Controls were injected with PBS. Each symbol represents the value for an individual mouse; horizontal lines indicate the mean value for each group. Data are pooled from two independent experiments. T test; **P<0,01 as compared with the 9602P-injected group. (**F**) *In vivo* effect of NK cell depletion on systemic bacterial dissemination in the spleen. Bacterial load was determined 18 h after infection into the ear with spores of the 9602P strain (3.05±0.29 log_10_ CFU per mouse). Data are pooled from two independent experiments.T test; *, *P*<0.05; **, *P*<0,01 as compared with the non-treated group.

NK cells patrol the host tissues to detect and react to any danger signal. No data are available on their involvement during *B. anthracis* infection. We therefore visualised *in vivo* NK cell recruitment into the initial infectious foci and the draining lymph node. Biphotonic imaging was performed on the infected ear of mice after inoculation with spores of an encapsulated toxin-deficient *B. anthracis* strain (due to confinement restrictions, these experiments could not be performed with encapsulated toxin-secreting strains); visualisation of the NK cells was carried out through intravenous adoptive transfer of CFSE-labeled purified NK cells in mice whose blood vascular system was labeled through injection of dextran-rhodamine (Figure 6A–D). During the first 5 h of infection, NK cells were observed circulating in the ear capillaries (#20 µm diameter) in both infected and control ears (Figure 6A). Occasionally, long-term (more than 6 sec) interaction of NK cells with the capillary endothelium could be observed in the infected ear (Figure 6B). At 24 h of infection, NK cells were observed in the infected ear tissue, outside the vascular bed in the collagen-rich tissue (as visualised in blue by the second harmonic), either close to the vascular lining (Figure 6C, top), or at a distance (Figure 6C, bottom). At this time of infection, NK cells began to be observed in the capsular sinus of the draining lymph nodes (Figure 6D). Thus, infection with spores of *B. anthracis* triggers recruitment of NK cells into the local site of infection and further migration into the subcapsular sinus of the draining lymph node, where these cells could exert their functions.

We then determine the early *in vivo* impact of toxin secretion by nascent bacilli on the local NK cell response. To this end, we characterised the recruitment of NK cells and macrophages into the draining lymph node at 24 h of infection with the encapsulated toxin-deficient *B. anthracis* strain, and its encapsulated derivatives expressing either ET or LT (Figure 6E). The total number of lymphoid cells and the percentage of F4/80^+^ cells and CD49b^+^ cells was increased in the draining lymph node of the encapsulated toxin-less infected mice, compared with the lymph node from PBS-injected mice (data not shown). Compared with an uninfected lymph node, the absolute number of CD49b^+^ cells (Figure 6E, left panel) and F4/80^+^ cells (Figure 6E, right panel) in the infected draining lymph nodes significantly increased by nine-fold and six-fold respectively; p<0.01). Production of either ET or LT during infection with the encapsulated strains 9602L(EF+PA+) or 9602C(LF+PA+) led to a significant decrease in the number of CD49b^+^ and F4/80^+^ cells in the infected lymph node, as compared with lymph nodes from mice infected with the encapsulated toxin-deficient strain (Figure 6E; p<0.01). These results clearly indicate that infection with *B. anthracis* spores is recognized by the innate immune system, leading to early recruitment of NK cells into the cutaneous tissue, and recruitment of NK cells and macrophages into the draining lymph node. Through the action of its toxins, *B. anthracis* blocked recruitment of both NK cells and macrophages.

To define the role of NK cells in *B. anthracis* infection, the effect of *in vivo* NK cell depletion was characterised. Accelerated systemic dissemination of the bacteria from the site of infection was observed, with early bacterial seeding of the spleen in NK-depleted mice *versus* control infected mice (Figure 6F). This result demonstrates the *in vivo* role of NK cells in controlling early *B. anthracis* dissemination.

## Discussion

In the present study, we have provided insight into the mechanisms of cytokine and cellular signaling that enable *B. anthracis* spores to efficiently drive IFN-γ production by NK cells. We also showed how *B. anthracis* toxins may allow the bacteria to avoid immune clearance by altering cytokine production and natural cytotoxicity.

The communication between NK cells and spore-activated macrophages was both cytokine- and contact-dependent and involved engagement of the NKG2D receptor. Our data indicate that macrophage-derived cytokines alone, or direct stimulation of NK cells by *B. anthracis* spores alone, are not sufficient to drive activation of NK cells, as assessed by CD69 and IFN-γ expression. Although IL-12 was secreted by spore-stimulated macrophages, NK cell activation did not occur when cellular interactions between NK cells and macrophages were prevented. Thus, cell-cell contact was a critical factor in macrophage/NK-cell interactions. Contact dependency involved at least engagement of the activating NKG2D receptor with one of its main ligands on the macrophage, RAE-1 [36], whose expression was upregulated upon spore stimulation. Stimulation with various TLR agonists leads to surface expression of RAE-1 on macrophages [37]. RAE-1-NKG2D interactions contribute to IFN-γ production and provide a molecular mechanism by which NK cells and infected macrophages communicate directly during an innate immune response to infection [38]. The requirement for direct contact between NK cells and macrophages for full NK cell activation might in part also be a manifestation of the dependency of NK cells on cytokine-mediated signals delivered when contact is established. For example, delivery to NK cells of IL-15 and IL-18 produced by macrophages and dendritic cells occurs in a synaptic manner, so that as soon as the cytokines are secreted, they are captured by the secreting or target cell [39], [40]. Secreted IL-15, for example, is immediately bound by the IL-15 receptor-α expressed on the surface of the accessory cells and is presented to NK cells in a cell-contact dependent manner [41]. Similarly, IL-18 is delivered to NK cells through cell contact with dendritic cells [39]. The IL-12 receptor may also localise at the contact zone between NK cells and macrophages [4], indicating that direct contact might be pivotal for efficient delivery of IL-12 to NK cells [42]. Our data are thus consistent with the strict contact-dependency of NK-cell activation, as these three cytokines are induced under spore stimulation and are strictly required for NK cell activation.

We demonstrated a predominant implication of IL-12 and IL-18 in NK-cell activation by *B. anthracis* spores, and to a lesser degree of IL-15; these cytokines were secreted by the spore-stimulated macrophages. However, individually, IL-12, IL-15, and IL-18 failed to induce effector responses in purified NK cells. IFN-γ production was almost abolished when both signals from IL-15 and IL-18 were simultaneously absent. These observations strongly suggest a synergistic mechanism of action of IL-12 with IL-15 and/or IL-18. Endogenous IL-15 produced by LPS-activated monocytes works in concert with IL-12 for optimal IFN-γ production by NK cells [43]. Several studies have shown that IL-18 responsiveness is dependent upon IL-12 and *vice versa* for T cells [44], [45]. IL-12 and IL-18 are considered important mediators of IFN-γ production by NK cells and T lymphocytes [46]. The molecular mechanism underlying the synergy between IL-18 and IL-12 may be explained in part by reciprocal modulation of cytokine receptor expression. Specifically, IL-18 upregulates IL-12R expression [47], whereas IL-12 upregulates expression of the IL-18R [48].

In our model, liberation of IL-18 was probably a primary event in response to *B. anthracis* spores. Short-term pretreatment with IL-18 prior to stimulation with spores fully restored IFN-γ production, even when IL-18 paracrine activity was later neutralized. In contrast, IL-12 was required for a longer time period to obtain full restoration of IFN-γ secretion. This may reflect differences in storage and delivery for these two cytokines. IL-18 is stored as a biologically inactive precursor in secretory organelles of the endolysosomal compartment and, upon stimulation, can be rapidly released into the extracellular milieu after cleavage by caspase-1 [49], [50], whereas IL-12 needs to be synthesised and secreted along the classical secretory pathway [51].

Finally, our data emphasise the key role of IL-12 which is produced by spore-activated macrophages, and demonstrate that it acts both on NK cells and macrophages. Absence of the IL-12 receptor on NK cells reveals the secondary key role of IL-18. For macrophages, the absence of the IL-12 receptor greatly decreased their capacity to provide the necessary accessory signals to NK cells. Indeed, a general and critical role of IL-12 in potentiating the accessory function of myeloid antigen presenting cells has been suggested by Grohmann *et al* [52]. IL-12 is thus not only a connective element between accessory cells and lymphocytes, but also a key molecule for programming macrophage and dendritic-cell functions [53], [54].

We demonstrated that the inflammatory response induced by *B. anthracis* spores requires MyD88-mediated signaling both on macrophages and NK cells. MyD88 is an adaptor protein which is essential for signaling downstream of many TLRs, and also of IL-18 and IL-1 receptors [55]. MyD88-implication in the NK cell response was thus expected, as IL-18 is crucial for NK cell activation by *B. anthracis* spores. On the other hand, MyD88-dependence of spore recognition by macrophages, leading to deficient IL-12 secretion and, by way of consequence, absence of NK cell activation, shows that it is pathogen-pattern based. However, the interactions between the components of the highly complex spore particle and TLRs or related receptors still need to be characterised in detail. Spore recognition most probably involve several receptors; knocking-out one or more TLRs has indeed been reported not to modify the biological response observed, either *in vitro* [10] or *in vivo* (Tod Merkel, personnal communication).

IL-18 is a proinflammatory cytokine that belongs to the IL-1 cytokine family. IL-18 and IL-1 are related in terms of structure, processing, receptor and signaling pathways [56]. Secretion of the active form of both cytokines is dependent on caspase-1 activation that is required for the processing of the IL-1 and IL-18 precursors. Release of mature IL-18 depends on concomitant activation of caspase-1 and TLR engagement by pathogen-derived agonists [56]. This suggests that spore recognition is able to deliver both activation signals. IL-18 amplifies the innate immune response by inducing the expression of cytokines and chemokines such as IL-1β, TNFα and IL-8. The macrophage cytokine response could thus be triggered both directly and indirectly by the spores.

*B. anthracis* toxins were highly efficient in subverting the innate immune response, triggered by *B. anthracis* spores through activation of macrophages and induction of IFN-γ secretion by NK cells. Even at very low doses, the toxins disrupted IFN-γ production by spore-stimulated splenocytes. They altered both contact-dependent and cytokine-dependent signaling. Both toxins reduced expression of RAE-1 on the surface of spore-stimulated macrophages, thus decreasing signaling through the activating receptor, NKG2D. Indeed, defects in the expression of RAE-1 molecules have been hypothesized to contribute to reduced NK cell function [57]. The mechanisms of inhibitory action for each toxin on cytokine secretion were different. LT targeted both macrophages and NK cells, whereas ET blocked the macrophage activating functions but did not affect the IFN-γ secretion capacity of NK cells. Impairment of IFN-γ production by ET depended mainly on inhibition of IL-18 production by macrophages, a primary event in NK cell stimulation. Basal IL-12 secretion by macrophages was not affected and was sufficient to drive normal amounts of IFN-γ secretion by NK cells when the external IL-18 concentration was restored.

A number of studies have addressed the effects of LT and ET on cytokine secretion and their consequences on immunity [18], [58], [59]. While LT inactivation of MKK signaling pathways leads to almost invariable inhibition of the innate immune response, ET-induced cAMP increase results in a complex immunomodulatory effect. LT inhibits, whereas ET differentially regulates the release of pro-inflammatory cytokines in macrophages and DCs [22], [60], [61].

Our results on ET-induced IL-12 inhibition are consistent with previous reports of an inhibitory effect of ET on macrophages and DCs [22]. Of note is our observation that ET has a dominant effect on IL-18 secretion. Secretion of IL–18 by activated macrophages depends on the protease caspase-1 that converts the IL-18 precursor to the mature and biologically active cytokine [62], [63]. Caspase-1 activation is induced by *B. anthracis* spores and has been suggested to play a critical role in host defences against *B. anthracis* infection *in vivo* [64]. LT has also been reported to induce caspase-1 activation which results in IL-1β and IL-18 intracellular processing [64]. However, the release of these cytokines occurs as a passive event, resulting from cell death and lysis [65].

NKT cells are a specialized subset of T lymphocytes sharing both T cell and NK cell markers with the capacity to recognise microbial glycolipid antigens [66]. However, their modes of recognition are distinct from NK cells and their functions are quite different [67]. The effect of LT on NKT cells has recently been explored after stimulation with their classical cognate ligand, α-galactosylceramide [23]. LT was reported to induce an anergy-like unresponsiveness in NKT cells following stimulation *via* their T cell receptor [23].

One of the best characterised functions of NK cells is their natural cytotoxic activity against virus-infected cells or cells undergoing tumor transformation [35]. The present study showed that both ET and LT directly altered the innate ability of NK cells to exert their natural cytotoxic function, both *in vitro* and, most importantly, *in vivo*. Furthermore, ET exerted a much stronger inhibitory effect than LT. EF -the enzymatic moiety of ET- is an adenylate cyclase leading to elevated concentrations of intracellular cAMP [16]. We demonstrated that similar *in vivo* inhibition of the NK cell cytotoxic activity was also induced by the CyaA toxin of *Bordetella pertussis*. Thus, as a bacterial adenylate cyclase, CyaA toxin produces the same end-effect *i.e.* increase of intracellular cAMP [68]. These data thus strongly suggest that inhibition of NK-cell cytotoxic activity by ET is mediated *via* the activation of cAMP downstream pathways. We thus provide the first demonstration that the ET subverting action leads to dissociation in NK-cell function, which strongly blocks natural cytotoxicity without affecting IFN-γ secreting capacity. Its high efficiency stresses the impact this toxin may exert on anthrax pathogenesis. NK cell-associated receptors have been implicated in certain autoimmune diseases [69], [70] and NK cells have been suggested to play a role in modifying T cell-mediated autoimmunity. As the cytotoxic activity of cytotoxic CD8 T cells shares at least in part, common mechanisms with NK cells [71], we believe these AMPc-elevating compounds could be of use to inhibit the deregulated or increased cytotoxic activities that underlie NK or CTL-dependent autoimmune pathology [72].

Our *in vivo* data show that NK cells are rapidly recruited into the cutaneous tissue infected by *B. anthracis*. Both NK cells and macrophages were detected early in the draining lymph node in the absence of toxin secretion. In contrast, production of either ET or LT during infection drastically inhibited this local immune response. The reduced inflammatory response could be related to the immunosuppressive activities of LT and ET [59]. By increasing the intracellular concentration of cAMP and cleaving MKKs, *B. anthracis* toxins have the potential to interfere with chemotactic signaling for neutrophils, T-cells and macrophages [14], [20], [73]. Considering that -in the context of anthrax infection- macrophages appear to afford protection to the host [14], [74], it is not surprising that *B. anthracis* has developed means of suppressing certain macrophage functions such as their migration to lymph nodes.

We furthermore demonstrated that NK cells controlled the infectious process, as *in vivo* NK cell depletion resulted in an increased bacterial dissemination to the spleen. Conceptually, NK cell depletion could mimick the consequences of NK cell inactivation by the toxins. Clearly, further experiments are needed to address how NK cells control *in vivo* spreading of bacteria during anthrax infection. In a previous study, we showed the central impact of ET on bacterial control of dissemination in the draining lymph node, both after cutaneous and inhalational infections [75]. The draining lymph node thus appears to be the key organ for delaying bacterial systemic dissemination. We postulate that this control most probably occurs through direct interactions between NK cells and accessory cells, resulting in IFN-γ production and macrophage activation of their bactericidal activity. NK cells respond to pathogens through both cytokine secretion and natural cytotoxicity. The relative impact of each NK function on the development of an infectious process depends on the type of infection [5]. Natural cytotoxicity has mainly been characterised against tumoral or virally-infected targets cells [76]. Direct natural cytotoxicity to infected cells in bacterial infections has rarely been reported, primarily with intracellular pathogens (*Mycobacterium sp.*, *Listeria monocytogenes*; [5]). As *B. anthracis* is an extracellular pathogen, we hypothesise that NK cell cytotoxic activity may potentially occur either at the initial infection step, when a proportion of the infecting spores are phagocytosed by accessory resident cells such as macrophages or dendritic cells - which could provide the necessary contact- and cytokine-dependent signaling to the NK cells, or, at a later stage of infection, through recognition by NK cells of cellular stress induced by the toxins. The potential role of NK cell cytotoxicity during *B. anthracis* infection remains to be explored in depth.

The present study is the first to investigate the direct modulation of NK cell functions, IFN-γ producing capacity and natural cytotoxicity, by *B. anthracis*, and their subversion by ET and LT. Our findings exemplify the delicate balance between stimulation of the initial host control mechanisms by *B. anthracis* spores and the bacterial evasion strategies to overcome these innate host defences. NK cells are important immune effectors for preventing microbial invasion and dissemination [77], performing their surveillance function and establishing intercellular communications at an early stage of infection. The model we propose hypothesises that interactions of macrophages and dendritic cells with the infecting spores and spore components would lead to NK cell activation and IFN-γ production, through a combination of signals derived from intercellular contacts with macrophages and from cytokines secreted by these cells. The accumulation of NK cells and macrophages in the appropriate cytokine environment of the infected lymph nodes will thereby amplify the inflammatory response. Such a positive feedback loop is likely to be important in the control and pathogenesis of anthrax. Furthermore, by secreting toxins, nascent *B. anthracis* bacilli will alter spore-induced contact-dependent signaling and cytokine production. This will prevent efficient immune-cell contacts and initiation of inflammation and inflammatory-cell recruitment into the infected draining lymph node, resulting in successful bacterial colonization and spreading of infection.

## Materials and Methods

### Ethics statement

All the animal experiments described in the present study were conducted at the Institut Pasteur according to the European Union guidelines for the handling of laboratory animals (http://ec.europa.eu/environment/chemicals/lab_animals/home_en.htm) and were approved by the animal care and use committee at the Institut Pasteur. All efforts were made to minimize suffering.

### Bacterial strains and mice

RPLC2 is a Sterne derivative that produces inactive lethal and edema factors mutated in their enzymatic site [78]. The 9602P (delta-*pagA*), 9602C (delta-*cya*) and 9602L (delta-*lef*) strains [75] are derivatives of the highly virulent natural human isolate 9602 [79]. RPLC2 spores were produced, purified on Radioselectan (Renografin 76%, Schering) and formaldehyde-inactivated as previously described [80], [81]. Formaldehyde-inactivated spores (FIS) were quantified using a Malassez counting chamber and inactivation was confirmed by plating on BHI agar. Heat inactivation was carried out as previously described [10]. *B. subtilis* strain SMY was a kind gift from Abraham L. Sonenshein (Department of Molecular Biology and Microbiology, Tufts University School of Medicine, Boston, USA) and spore inactivation was performed as for *B. anthracis*. The germination-deficient mutant on the 7702 background was constructed by inactivation of the *cwlD*, *sleB* and *cwlJ1* genes through double crossing-over insertion of antibiotic cassettes (spectinomycin, erythromycin and kanamycin respectively) using techniques as previously described [82] (Manuel Lopez-Vernara, Fabien Brossier and Michèle Mock, unpublished results). Germination was decreased by at least 6 log_10_ on BHI agar. The inoculum used in the *in vitro* cell infection assays (2×10^6^ spores per well, as assessed by counting in a Malassez chamber) did not give rise to any CFU upon numeration.

Six- to 10-week-old C57BL/6 and FVB female mice were purchased from Charles River (L'Arbresle, France). CD1d-deficient C57BL/6 mice lacking both CD1d1 and CD1d2 [83] were a kind gift from Dr Claire-Lise Forestier (G5 Parasite Virulence, Institut Pasteur, Paris, France). β2m-deficient C57BL/6 mice, B6.129P2-*B2m^tm1Unc^*/J (Jackson Laboratory), were a kind gift from Dr Matthew Albert (Dendritic Cell Immunobiology, Institut Pasteur, Paris, France). The IL-12Rβ2-deficient and IL-12p35/p40-deficient C57BL/6 mice (both from Jackson Laboratory) were kindly provided by Dr Selina Keppler (University Hospital Freiburg, Institute of Medical Microbiology and Hygiene, Freiburg). The MyD88-deficient mice obtained from the laboratory of Shizuo Akira were backcrossed eight times to the C57BL/6 background [55] and bred in the central animal facility of the Pasteur Institute. The animals were housed in the animal facilities of the Institut Pasteur licensed by the French Ministry of Agriculture and complying with the European regulations. The protocols were approved by the safety committee at the Institut Pasteur according to the standard procedures recommended by the animal care and use committee at the Institut Pasteur.

### Cell activation assay and immunodetection of cytokines

Single spleen cell suspensions were prepared by mechanical disruption on a cell strainer (70 µm pore diameter, BD Biosciences, Bedford, USA) in Dulbecco's D-PBS (Invitrogen). Red blood cells were lysed using Hemolytic Gey's Solution as previously described [10]. NK cells were purified using the EasySep Mouse panNK (CD49b) Positive Selection Kit (StemCell Technologies, Vancouver, British Columbia, Canada) according to the manufacturer's protocol. Flow cytometry analysis showed the purity of NK cells to be more than 90%. Further purification (>98%) was performed using a MoFlo cell sorter (Beckman-Coulter). Bone marrow-derived macrophages (BMDMs) were obtained from the femur of mice after differentiation for 8 to 10 days in RPMI complete medium supplemented with 20 ng/ml M-CSF (PeproTech, Levallois-Perret, France) on bacterial petri dishes. BMDMs were mature as assessed by expression of the F4/80 surface marker (consistently over 90% F4/80 positive). Cell enumeration and viability (>90%) was routinely assessed by acridine orange/propidium iodide staining.

All cell cultures were carried out in RPMI 1640 medium+GlutaMAXTM I (Invitrogen, Cergy-Pontoise, France) supplemented with 10% fetal calf serum (FCS; BioWest, Nuaillé, France), 100 µg/ml penicillin/streptomycin (Invitrogen), and 50 µM 2-mercaptoethanol (Invitrogen). All reactivation conditions were performed in triplicate.

Cell activation was performed by incubating either 2×10^5^ splenocytes, or 2×10^5^ purified NK cells with 2×10^5^ BMDM, with 2×10^6^ spores (or polystyrene latex beads −1.1 µm mean particle size-, Sigma, Aldrich) in a final volume of 200 µl in 96-well tissue culture plates (TPP, Trasadingen, Switzerland) for 1 to 3 days. Direct stimulation of purified NK cells was performed by adding to the culture medium endotoxin-free mouse rIL-12 (10 ng/ml, BD Pharmingen) and endotoxin-free mouse rIL-18 (20 ng/ml, MBL International) for 18 h. In some experiments, NK cell activation was performed in 96-well Transwell plates (0.4 µm pore diameter; Corning Costar, NY); BMDMs (3.1×10^5^ cells in 235 µl) mixed with FIS (3.1×10^6^) were seeded into the outer chamber; CD49b^+^ NK cells (3.1×10^5^ cells in 75 µl) were added either directly to the outer chamber, or placed into the inner chamber along with or without 3.1×10^6^ FIS. Cytokine and receptor function were blocked by addition of the following azide-free, low-endotoxin anti-mouse cytokine or receptor-specific mAbs: rat anti-mouse IL-12p40/p70 (5 µg/ml, clone C17.8, BD Pharmingen) and its isotype control (clone R35-95, BD Pharmingen), rat anti-mouse IL-18 (10 µg/ml, clone 93-10C, MBL International Corporation) and its isotype control (clone eBRG1, eBioscience), goat anti-mouse IL-15Rα polyclonal (10 µg/ml, R&D Systems), armenian hamster anti-mouse NKG2D (10 µg/ml, clone C7, eBioscience) and its isotype control (clone eBio299Arm, eBioscience), rat anti-mouse RAE-1 (5 µg/ml, clone 199205, R&D Systems) and its isotype control (clone R35-95, BD Pharmingen), rat anti-mouse IFN-α (5 µg/ml, clone RMMA-1, PBL Biomedical Laboratories), rat anti-mouse IFN-β (5 µg/ml, clone RMMB-1, PBL Biomedical Laboratories), goat anti-mouse NKp46 polyclonal (10 µg/ml, R&D Systems), rat anti-mouse IL-10 (10 µg/ml, clone JES5-2A5, BD Pharmingen), armenian hamster anti-mouse TNF-α (10 µg/ml, clone TN3-19.12, Sigma).

For analysis of the effects of the toxins, splenocytes were pre-treated before stimulation, for 1 h with rPA (250 ng/ml) and/or either rLF (0.01–100 ng/ml), -both a kind gift from Dr Bassam Hallis, HPA, Porton Down, UK-, or rEF (0.01–100 ng/ml) -a kind gift from Pr Wei-Jen Tang, University of Chicago, Chicago, USA. Purified CD49b^+^ NK cells were pre-treated before stimulation for 1 h with PA (250 ng/ml) and/or either LF (0.5–100 ng/ml) or EF (10–100 ng/ml), or PA (2500 ng/ml) and EF (500–1000 ng/ml). NK cell viability after toxin treatment was analyzed by using Live/Dead Cell Staining Kit (Invitrogen) and their metabolic activity was assessed by the CellTiter 96 AQueous One Solution Cell Proliferation Assay (MTS) (Promega).

In all cases, cell-free supernatants were removed at specific time points and frozen at −20°C before subsequent ELISA analysis for IFN-γ or IL-12. When mentioned, conditioned supernatants from FIS-activated BMDM were collected after a 24-h stimulation, transferred on purified CD49b^+^ NK cells and incubated for a further 48 h. ELISA was performed as previously described [10] using the following antibody pairs and protein standards (all from BD Pharmingen, Le-Pont-de-Claix, France): for IL-12p40/p70, capture with mAb clone C15.6, detection with mAb clone C17.8, standard recombinant mouse IL-12 (p70); for IFN-γ, capture with mAb clone R4-6A2, detection with mAb clone XMG1.2, standard recombinant mouse IFN-γ.

### Flow cytometry

Cell staining was performed with the following mAbs: anti-mouse CD69-fluorescein isothiocyanate (FITC) (clone H1.2F3) (BioLegend, San Diego, USA) and its FITC-conjugated isotype control (Caltag laboratories), CD49b- phycoerythrin (PE) (clone DX5) (BD Bioscience, San Diego, CA), IFN-γ-FITC (clone XMG1.2) (BD Bioscience, San Diego, CA) and its FITC-conjugated isotype control (BioLegend), F4/80-allophycocyanine (APC) (clone BM8) (BioLegend), pan-RAE-1-PE (clone 186107) (R&D Systems) and its PE-conjugated isotype control (Caltag Laboratories). To obtain a single-cell suspension, collected cells were pre-treated in 5 mM EDTA/PBS to dissociate cell aggregates. Cells were subsequently washed and blocked for 10 min with anti-CD16/CD32 mAb (BioLegend) in FACS buffer (Dulbecco's PBS, 2% heat-inactivated FBS, 10 mM sodium azide) and then labeled with the appropriate antibodies. Dead cells were excluded during acquisition through staining with LIVE/DEAD Fixable Dead Cell Stain Kit according to the manufactor's procedure (Invitrogen). For intracellular cytokine staining [84], cells were incubated with brefeldin A (5 µg/ml, Sigma, Aldrich) for the last 4 h of activation with the spores. After labeling with DX5-PE mAb, fixation in 2% paraformaldehyde and permeabilisation in 0.5% saponine, the cells were stained with anti-IFN-γ-FITC mAb for 30 min. Cell acquisition was performed using a FACSCalibur flow cytometer (BD Bioscience). NK cells were gated by their light scattering properties -forward (FSC) and side (SSC) scatter- that distinguished them from the macrophages (Figure S1A). Isotype controls Abs were used for each staining combination. A minimum of 10,000 events was acquired for analysis. Data were acquired and analyzed using CELLQUEST software (BD Bioscience). Figures were derived by free WinMDI Software (Version 2.8, Bio-Soft Net; WinMDI software [http://en.bio-soft.net/other/WinMDI.html]).

### CFSE-based *in vitro* cytotoxicity assay

Natural cytotoxicity assay was performed as previously described [85]. Briefly, the mouse lymphoma YAC-1 target cells were labeled with 2.5 µM CFSE (Sigma, Aldrich) at 2×10^6^ cells/ml for 8 min at room temperature. After dilution by 1∶5 in complete medium and 2 washing steps, the CFSE-labeled target cells were resuspended in complete medium (4×10^4^ cells/ml, 100 µl/tube) and mixed with positively selected CD49b^+^ cells at an effector/target cell ratio (E/T ratio) of 50∶1 (final volume: 200 µl) in 5 ml Falcon round-bottom tubes (BD Bioscience). When necessary, CD49b^+^ cells were treated for 4 h with PA (250 ng/ml) and/or either LF (100 ng/ml) or EF (100 ng/ml), and then washed prior to mixing. The cells were then incubated in the presence of 10 ng murine rIL-2 (BioLegend) in a humidified atmosphere of 5% CO2 at 37°C for 18 h. Cytotoxicity was then assessed by flow cytometry analysis after propidium iodide labeling. Negative controls were CFSE-labeled target cells without NK cells.

For data analysis, the CFSE-stained target cells were gated (R1) on SSC/FL1(CFSE) parameters and analyzed on FL1(CFSE)/FL3(Propidium iodide). For each sample 4000 events of R1 were collected. The percentage of specific target cell death was calculated as follows: [(dead CFSE-positive targets in the sample (%)−spontaneously dead CFSE-positive targets (%))/(100−spontaneously dead CFSE-positive targets)]×100.

### *In vivo* cytotoxicity assay

Spleen cells from C57BL/6 and β2m^−/−^ C57BL/6 mice were labeled with 2 µM (C57BL/6) or 5 µM (β2m^−/−^ C57BL/6) CFSE, and equal cell numbers (10^6^; “injected mix”) were co-injected intravenously into C57BL/6 mice recipients, either untreated or injected intravenously 8 h prior to cell inoculation with (i) LF (7.5 µg) or EF (7.5 µg) and PA (20 µg) or (ii) CyaA or the inactive mutant CyaE5 (15 µg) of *Bordetella pertussis* (a kind gift from Dr Daniel Ladant, Biochimie des Interactions Macromoléculaires, Institut Pasteur, Paris, France). Control mice received either EF or PA alone. *In vivo* NK cell depletion was performed by intravenous injection of 200 µg anti-NK1.1 mAb (clone PK136, Serotec, Oxford, United Kingdom). *In vivo* NK cell activation was performed by intravenous injection of 100 µg poly:(IC) (Invivogen). Single spleen cell suspensions were prepared 16 h later and the CFSE positive cell population was acquired by FACS as described above. The ratio of CFSE high *versus* CFSE low cells was determined and specific lysis was calculated as described [86]: 100×[1−(ratio of injected mix/recovery ratio)], where ratio = % CFSE low/% CFSE high.

### MAPK phosphorylation and cAMP assays

Purified NK cells (10^6^ cells) were incubated with PA (250 ng/ml), and/or LF (100 ng/ml) for 2 h at 37°C. Recombinant IL-12 (50 ng/ml) and IL-18 (100 ng/ml) were then added for 10 min, cells were washed twice with cold PBS and lysed on ice for 30 min with a total protein extraction buffer (20 mM Tris-HCl, pH 7.5, 150 mM NaCl, 1 mM sodium EDTA, 1 mM EGTA, 1% Nonidet P-40, 1% sodium deoxycholate, 2.5 mM sodium pyrophosphate, 1 mM β-glycerophosphate, 1 mM Na3VO4, 1 µg/ml leupeptin) containing Complete protease and phosphatase inhibitor tablets as specified by the manufacturer (Roche Diagnostics). Total protein (50 µg as determined by the Bradford assay, Bio-Rad Laboratories) was resolved on 10% SDS-PAGE gels and transferred to nitrocellulose membranes. Membranes were blocked in TBS containing 5% non-fat dried milk and 0.1% Tween 20. Protein detection was performed with polyclonal Abs directed against phospho-p38 MAPK, phospho-p42/44 (ERK 1/2) MAPK, phospho-JNK or total p42/44 (ERK 1/2) MAPK (all from Cell Signaling Technology). Total p42/44 (ERK 1/2) MAPK was used as loading control as previously described [87]. Bands were visualized with appropriate secondary HRP-conjugated Abs and SuperSignal West Pico chemiluminescent substrate (Pierce). When reprobed, membranes were first stripped by incubating in a stripping buffer (Gene Bio-Application Ltd.).

To determine the cAMP response generated by edema toxin, purified CD49b^+^ NK cells (10^6^ cells) were incubated with PA (0.25 µg/ml) and EF (0.05–0.1 µg/ml), or PA (2.5 µg/ml) and EF (0.5–1 µg/ml) at 37°C for 1 h and then stimulated with rIL-12 (10 ng/ml) and rIL-18 (20 ng/ml). After 2 h of incubation, the culture medium was discarded and the cells were lysed with 0.1 M HCl. After centrifugation, the supernatants were collected and immediately stored at −80°C before analysis. Intracellular cAMP concentration was determined using a commercial cAMP EIA kit (Cayman Chemical, Ann Arbor, MI, USA) according to the manufacturer's instructions.

### Isolation of lymph node cells and flow cytometry

FVB mice were infected into the ear dermis with spores of the non-toxinogenic encapsulated 9602P (EF+LF+) strain, or the 9602L (EF+PA+) or 9602C (LF+PA+) strains. Cutaneous infections were performed under light anesthesia by injecting 2.91±0.03 log_10_ spores per mouse in 10 µl of PBS into the dermis of the left ear as previously described [88]. 24 h later the cervical lymph nodes draining the infected site were excised, placed into ice-cold saline solution and mechanically dissociated to obtain single-cell suspensions. Control lymph nodes were obtained from PBS-inoculated ears. Cell labeling was performed with APC-conjugated anti-F4/80 (BioLegend), anti-CD4-FITC (BD PharMingen), anti-CD8-PE (BD PharMingen), and PE-conjugated DX5 (eBioscience). Flow cytometry acquisition was performed as described above.

### Effect of NK cell depletion upon *B. anthracis* infection

NK-cell depletion was performed in C57BL/6 mice by intraperitoneal injection of 200 µg of anti-NK1.1 antibody (clone PK136, Serotec, Oxford, United Kingdom) at 2 days and 1 day before infection. Depletion of NK cells (>95%) was verified in the spleen by flow cytometry. Control mice received an injection of 200 µg isotype-matched antibody (Sigma). Cutaneous infection in the ear was performed by injection of 3.05±0.29 log_10_ spores of the 9602P strain. Spleens were removed aseptically 18 h post-inoculation and homogenized in 5 ml of PBS. The bacterial load in the resulting suspensions was determined by plating 100 µl of 10-fold dilutions onto BHI agar plates and is expressed as log_10_ CFU per spleen.

### Two-photon excitation fluorescence *in vivo* imaging

C57BL/6 mice were subcutaneously infected with 2×10^3^ spores of the non-toxigenic encapsulated *B. anthracis* strain in 10 µl of PBS into the external face of the right ear, while the same volume of PBS was injected in the left ear. Prior to imaging, 100 µg of rhodamine B 10 kD-dextran (Sigma) were administrated by intravenous injection. Negatively selected NK cells (Negative selection mouse NK cell enrichment kit, StemCell Technologies) were labeled with 5 µM of CFSE and were injected intravenously (10^6^ per mice) 4 h after infection. Ketamine-xylazine anesthetized mice were placed under the microscope with their ears maintained between cover glasses. The cervical lymph nodes draining the infected and the PBS-injected control ears were harvested from the recipient mice 24 h after infection, fixed with tissue glue to the plastic chamber containing PBS, and sequentially imaged. Two-photon excitation fluorescence (TPEF) imaging was performed using a LSM 710 Zeiss microscope. The excitation wavelength is 854 nm, allowing epicollection of second-harmonic generated signal (SHG) at 427 nm quasi specific of collagen. Other epicollected signals were intravenous rhodamine B and CFSE-stained NK cells fluorescence. Image acquisition and analysis were performed by using ZEN 2008 software (Zeiss).

### Statistical analysis and software

Statistical analysis was performed using Graphpad Prism software. Unless otherwise noted, results are expressed as mean values ± standard deviation. The student's t-test was used to determine significance (*P*<0.05).

## Supporting Information

Figure S1 **Controls for NK cell activation, cytokine secretion and natural cytotoxicity.** (**A**) FACS analysis of surface CD69 and intracellular IFN-γ expression in CD49b^+^ cells in the presence of spore-stimulated BMDMs. NK cells were gated by their light scattering properties -forward (FSC) and side (SSC) scatter- that distinguished them from the macrophages (left panels), then dead cells were excluded along the FL4 channel through staining with LIVE/DEAD Fixable Dead Cell Stain Kit (Invitrogen). Isotype-matched controls Abs were used for each staining combination (see Materials and Methods). (**B**) Effect of neutralization of IFN-α, IFN-β, TNF-α, IL-10 and NKp46 on IFN-γ production by splenocytes. (**C**) Cell viability of splenocytes 18 h after incubation with PA+LF, or PA alone, LF alone compared with untreated splenocytes, as assessed by Live/Dead staining. (**D**) Absence of effect on the elimination of MHC class I-deficient β2m−/− splenocytes for the control groups receiving PA alone, EF alone or LF alone (see Figure 5B).
(TIF)
