# Supplementary figures and images for: Mechanisms of NK Cell-Macrophage Bacillus anthracis Crosstalk: A Balance between Stimulation by Spores and Differential Disruption by Toxins
Source: PLoS Pathog. 2012 Jan 12;8(1):e1002481. doi: 10.1371/journal.ppat.1002481 (PMC3257302; doi:10.1371/journal.ppat.1002481)

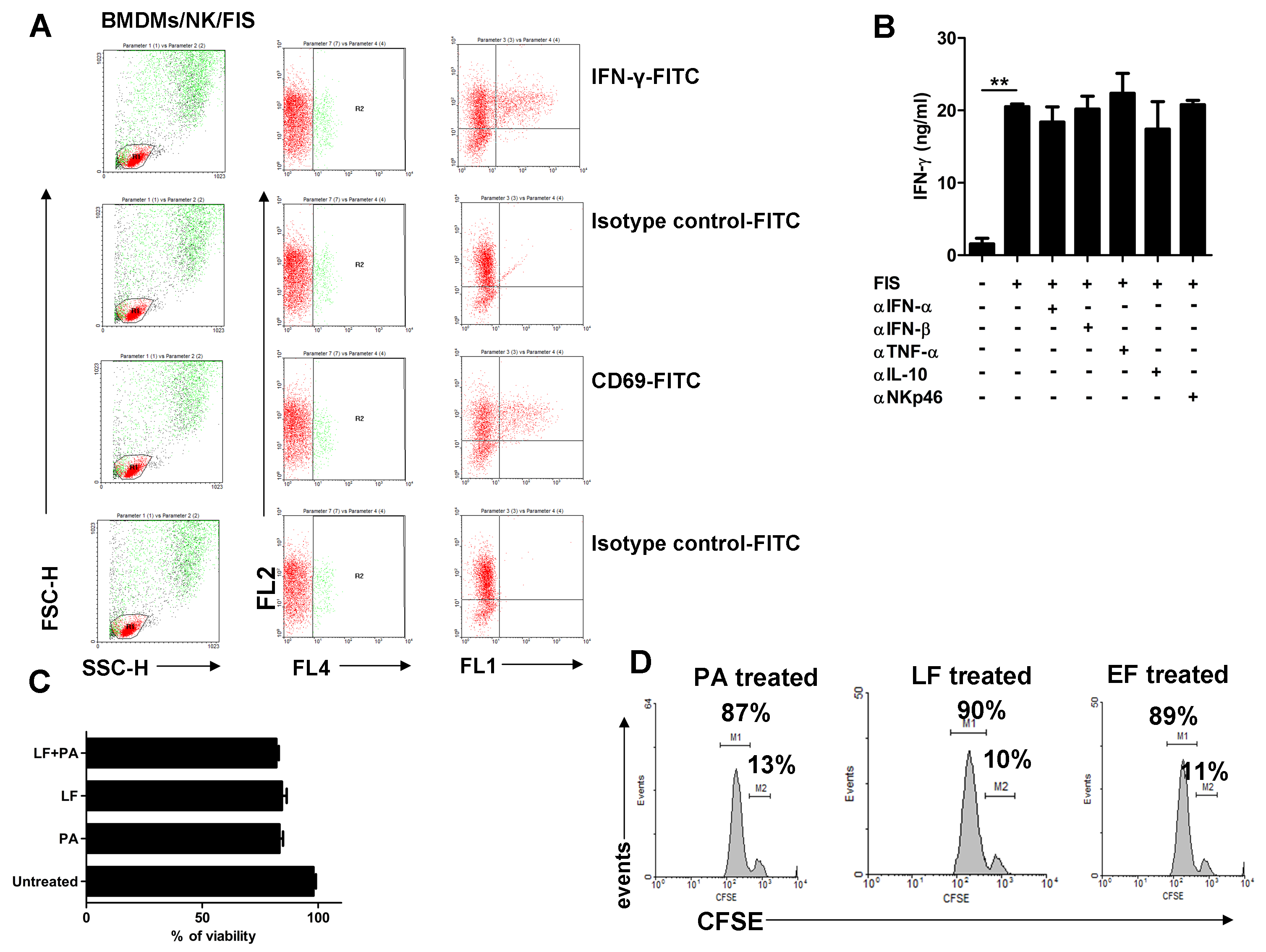

Supplement: Figure S1 — Controls for NK cell activation, cytokine secretion and natural cytotoxicity. (A) FACS analysis of surface CD69 and intracellular IFN-γ expression in CD49b+ cells in the presence of spore-stimulated BMDMs. NK cells were gated by their light scattering properties -forward (FSC) and side (SSC) scatter- that distinguished them from the macrophages (left panels), then dead cells were excluded along the FL4 channel through staining with LIVE/DEAD Fixable Dead Cell Stain Kit (Invitrogen). Isotype-matched controls Abs were used for each staining combination (see Materials and Methods). (B) Effect of neutralization of IFN-α, IFN-β, TNF-α, IL-10 and NKp46 on IFN-γ production by splenocytes. (C) Cell viability of splenocytes 18 h after incubation with PA+LF, or PA alone, LF alone compared with untreated splenocytes, as assessed by Live/Dead staining. (D) Absence of effect on the elimination of MHC class I-deficient β2m−/− splenocytes for the control groups receiving PA alone, EF alone or LF alone (see Figure 5B). (TIF) [file ppat.1002481.s001.tif]
